# Renal function and adverse clinical events in anticoagulated patients with atrial fibrillation: insights from the GLORIA-AF Registry Phase III

**DOI:** 10.1007/s11239-025-03067-5

**Published:** 2025-02-09

**Authors:** Yang Liu, Steven Ho Man Lam, Giulio Francesco Romiti, Bi Huang, Yang Chen, Tze Fan Chao, Brian Olshansky, Kui Hong, Menno V. Huisman, Gregory Y. H. Lip, on behalf of the GLORIA-AF Investigators

**Affiliations:** 1 Liverpool Centre for Cardiovascular Science at University of Liverpool, Liverpool John Moores University and Liverpool Heart & Chest Hospital, Liverpool, UK; 2 Department of Cardiovascular Medicine, The Second Affiliated Hospital, Jiangxi Medical College, Nanchang University, Nanchang, Jiangxi China; 3 Department of Cardiology, The First Affiliated Hospital of Chongqing Medical University, Chongqing, China; 4 Department of Translational and Precision Medicine, Sapienza University of Rome, Rome, Italy; 5 Division of Cardiology, Department of Medicine, Taipei Veterans General Hospital, Taipei, Taiwan; 6 Institute of Clinical Medicine, and Cardiovascular Research Center, National Yang-Ming University, Taipei, Taiwan; 7 Division of Cardiology, The University of Iowa, Iowa City, IA USA; 8 Department of Genetic Medicine, The Second Affiliated Hospital of Nanchang University, Nanchang, Jiangxi China; 9 Jiangxi Key Laboratory of Molecular Medicine, The Second Affiliated Hospital of Nanchang University, Nanchang, China; 10 Department of Medicine – Thrombosis and Hemostasis, Leiden University Medical Center, Leiden, The Netherlands; 11 Department of Clinical Medicine, Aalborg University, Aalborg, Denmark

**Keywords:** Atrial fibrillation, Oral anticoagulant, Vitamin K antagonists (VKA), Non-vitamin K antagonist oral anticoagulants (NOACs), Renal function

## Abstract

Renal function, assessed by creatinine clearance (CrCl), affects the efficacy and safety of oral anticoagulant (OAC) therapy in patients with atrial fibrillation (AF). To investigate the association between CrCl and the risk of clinical adverse events and compare the safety profiles of vitamin K antagonists (VKA) and non-vitamin K antagonist oral anticoagulants (NOAC). Patients with newly diagnosed AF (< 3 months before baseline visit) were collected from the prospective Global Registry on Long-Term Oral Anti-Thrombotic Treatment in Patients with Atrial Fibrillation (GLORIA-AF) registry Phase III. Clinical events assessed included the composite outcome (all-cause death, thromboembolism, and major bleeding), cardiovascular (CV) death, myocardial infarction (MI), and other single outcomes. 10,594 AF patients (mean age 70.35 ± 9.92 years; 55% male; 73% on NOAC) were included. Increasing CrCl was associated with decreased risks of all cause death, composite outcomes and CV-death with in patients with CrCl < 80 mL/min. Multivariate Cox models indicated that compared to VKA, NOAC was associated with lower risks of all cause death (adjusted hazard ratio [aHR] 0.68, 95% CI 0.58–0.78), composite outcomes (aHR 0.77, 95% CI 0.69–0.86), CV-death (aHR 0.70, 95% CI 0.56–0.87), and major bleeding (aHR 0.74, 95% CI 0.61–0.91) in AF patients. For CrCl < 30 mL/min, lower risks of all-cause death, composite outcomes and CV death were related to NOAC therapy. In this large prospective global registry, NOACs were associated with better outcomes compared with VKA for patients with normal or impaired renal function.

**Graphical abstract:**

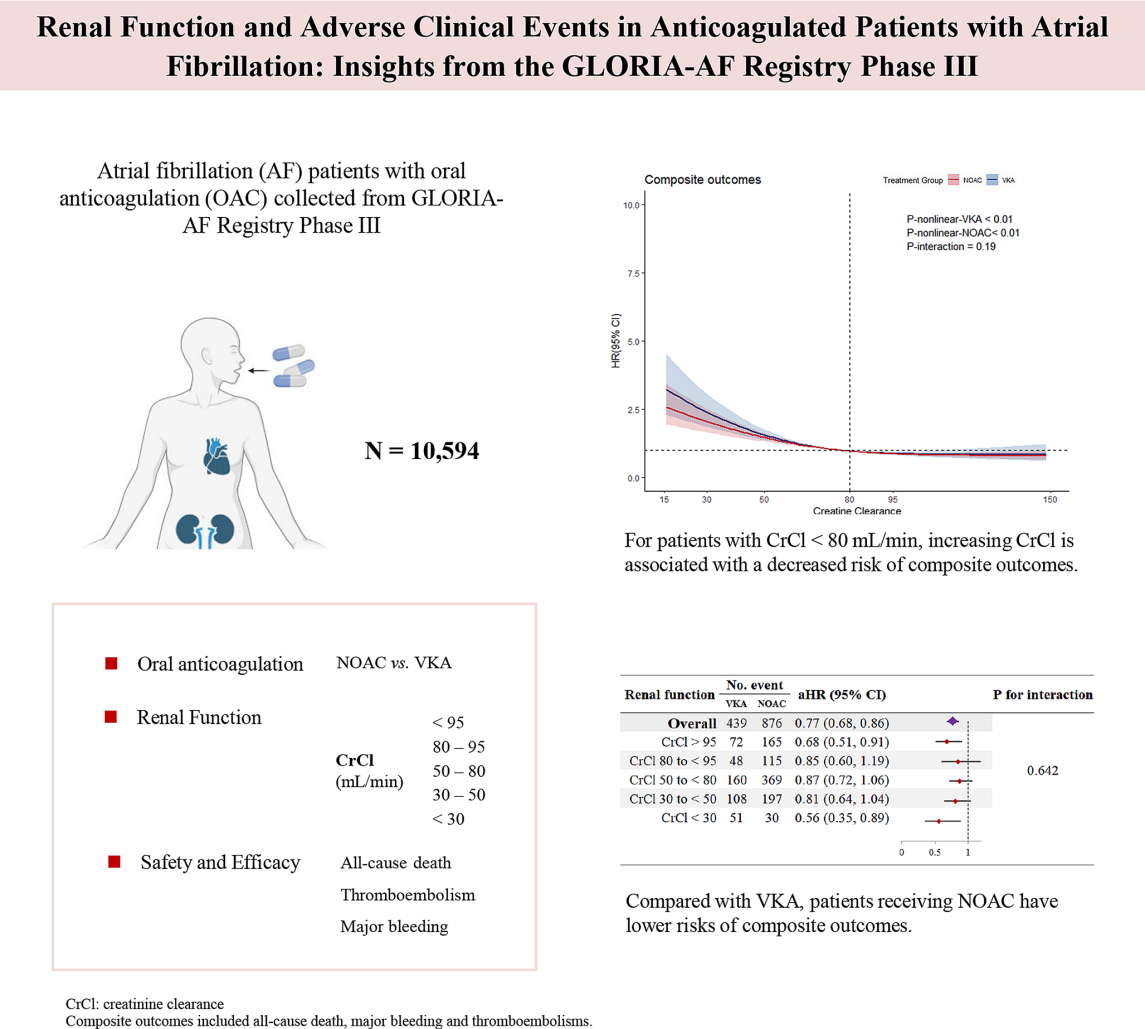

**Supplementary Information:**

The online version contains supplementary material available at 10.1007/s11239-025-03067-5.

## Introduction

Atrial fibrillation (AF) significantly increases the risk of stroke, leading to a substantial and growing burden on modern healthcare services [1]. To reduce stroke risk, the non-vitamin K antagonist oral anticoagulants (NOACs) have emerged as the preferred choice compared to the vitamin K antagonists (VKA), especially for patients newly initiating anticoagulation therapy [2].

NOACs exhibit varying degrees of renal dependency for excretion, making the assessment of renal function essential for determining appropriate dosing in line with guidelines [3, 4], typically using the Cockcroft-Gault formula to estimate creatinine clearance (CrCl) [5]. In patients with AF, chronic kidney disease (CKD) is independently associated with increased major bleeding and all-cause mortality [6, 7]. Consequently, considerable attention has been directed toward the safety of OAC therapy across varying renal functions, which includes ensuring appropriate dosing, evaluating drug persistence, and monitoring renal function changes over time to guide clinical prescribing decisions effectively [8]. Rather than retrospective monocentre data, more prospective global multicentre data on renal function in anticoagulated patients are needed.

The Global Registry on Long-Term Oral Anti-Thrombotic Treatment in Patients with Atrial Fibrillation (GLORIA-AF) registry is a multicentre observational study enrolling up to 56,000 newly diagnosed nonvalvular AF patients in nearly 50 countries [9]. We assessed the effect of renal function on the occurrence of clinical adverse events by comparing NOAC and VKA treatment, in this prospective global registry.

## Methods

### Study population

GLORIA-AF included patients with newly diagnosed atrial fibrillation (< 3 months before baseline visit). The study design has been previously reported [10]. During phase III (January 2014-December 2016), patients with or without OAC prescriptions were recruited with 3-year follow-up with scheduled visits at 6, 12, 24 and 36 months. Recruited patients were treated based on local clinical practice, with treatment choices determined by treating physicians. During follow-up, all major events, concomitant disease, and treatment were recorded.

At baseline, the following data were collected: age, sex, race, body mass index (BMI), smoking and drinking status, type of AF (paroxysmal, persistent, permanent), CHA_2_DS_2_-VASc score, comorbidities (hypertension, diabetes, hyperlipidemia, chronic artery disease, congestive heart failure, Transient Ischemic Attack (TIA), peripheral artery disease, chronic obstructive pulmonary disease), and history of clinical events (history of thromboembolism, stroke, bleeding), OAC therapy and antiplatelet therapy. Patients with missing renal function (serum creatinine), OAC therapy and clinical outcomes were excluded.

### Renal function estimation and classification

Creatinine clearance, used to estimate renal function, was calculated according to Cockroft-Gault in males: CrCl (mL/min)=(140-age)*weight(kg)/72*serum creatinine(*0.85 if females) [11]. The population was divided into five groups by baseline CrCl: >95, 80 to < 95, 50 to < 80, 30 to < 50, <30 mL/min. To explore the relationships between OAC therapy and renal function, patients were then sub-grouped by OAC treatment, including NOAC versus VKA, or individual NOAC (apixaban, rivaroxaban, dabigatran, edoxaban) versus VKA.

### Clinical adverse events and endpoints

Myocardial infarction (MI) was defined as the development of significant Q-waves in at least 2 adjacent electrocardiogram leads or met criteria reported in previous study [12]. Major bleeding was defined according to the International Society of Thrombosis and Haemostasis classification [13]. Stroke was described as an acute onset of a focal neurological deficit of presumed vascular origin lasting for ≥ 24 h, or resulting in death, including ischemic stroke, haemorrhagic stroke, and uncertain classification strokes. Thromboembolism (TE) was defined as a composite of ischemic stroke, transient ischemic attack, and non-central-nervous-system atrial embolism.

We examined clinical endpoints including a composite outcomes, defined as ‘all-cause death, TE and major bleeding’. We also examined individual outcomes including all-cause death, major bleeding, cardiovascular (CV) death, MI, stroke and TE.

### Statistical analysis

Continuous variables were represented by the mean (± standard deviation (SD)) and were compared by student’s t-test while categorical variables were represented by frequencies and percentages (n (%)) and were compared by Pearson’s Chi-squared test. The cumulative events between NOAC and VKA were compared using the Chi-squared test across various levels of renal function, respectively.

Multivariable Cox proportional hazard model was applied to compare the association between the adverse outcomes and OAC treatment at different levels of CrCl. Hazard ratio (HR) and 95% confidence intervals (CI) were calculated for indicators by comparing the VKA group and NOAC group in five levels of CrCl, respectively, and shown by table and forest plot. Restricted cubic spline (RCS) curves were used to explore the nonlinear relationship between CrCl and adverse clinical events and this relationship was compared between VKA and NOAC treatment [14]. Reasonable knots were selected according to the Akaike Information Criterion (AIC). The knots in outcomes of all-cause death, composite outcomes, CV death, major bleeding, MI, and stoke were set as 3, and TE was set as 4. Reference was set as CrCl = 80 mL/min.

Interaction analyses were used to assess if the association between renal function and various clinical outcomes are modified by (1) different OAC treatments; and (2) different ethnic groups (Asian/non-Asian). Multivariable models were adjusted by age, sex, race, BMI, smoking and drinking status, comorbidities (coronary artery disease, congestive heart failure, chronic obstructive pulmonary disease, peripheral artery disease, diabetes, hypertension), prior TE, previous bleeding, and any antiplatelet drug use.

The statistical analysis for this study was conducted using R (version 4.3.1, R Core Team 2020, Vienna, Austria). Results with *P* < 0.05 were deemed to be statistically significant.

## Results

### Baseline characteristics

A total of 10,594 patients with AF (age 70.35±9.92 years, 55% males, 56% paroxysmal AF) were included and divided into five groups by CrCl: ≥ 95, 80 to < 95, 50 to < 80, 30 to < 50 and < 30 mL/min (Table 1). Overall, 2,797 (26%) patients received apixaban, 2,273 (21%) dabigatran, and 2,485 (23%) rivaroxaban.

**Table 1:** Baseline characteristic of patients with atrial fibrillation in different levels of creatinine clearance

Characteristic	Overall *N* = 10,594	CrCl ≥ 95 mL/min *N* = 3,046	CrCl 80 to < 95 mL/min *N* = 1,730	CrCl 50 to < 80 mL/min *N* = 4,224	CrCl 30 to < 50 mL/min *N* = 1,372	CrCl < 30 mL/min *N* = 222
**Anticoagulation therapy**						
VKA	2,859 (27%)	725 (24%)	452 (26%)	1,147 (27%)	421 (31%)	114 (51%)
Apixaban	2,797 (26%)	807 (26%)	440 (25%)	1,061 (25%)	427 (31%)	62 (28%)
Dabigatran	2,273 (21%)	635 (21%)	420 (24%)	981 (23%)	216 (16%)	21 (9.5%)
Rivaroxaban	2,485 (23%)	839 (28%)	386 (22%)	948 (22%)	287 (21%)	25 (11%)
Edoxaban	180 (1.7%)	40 (1.3%)	32 (1.8%)	87 (2.1%)	21 (1.5%)	0 (0%)
**Age**,** years old**						
Mean (SD)	70.35 (9.92)	62.50 (9.42)	68.80 (8.01)	73.36 (7.53)	79.22 (6.21)	78.20 (8.81)
Median (25%, 75%)	71.00 (65.00,77.00)	64.00 (57.00,69.00)	69.00 (64.00,74.00)	74.00 (69.00,79.00)	80.00 (75.00,84.00)	81.00 (74.00,85.00)
**Sex**,** n (%)**						
Male	5,783 (55%)	2,048 (67%)	990 (57%)	2,152 (51%)	495 (36%)	98 (44%)
Female	4,811 (45%)	998 (33%)	740 (43%)	2,072 (49%)	877 (64%)	124 (56%)
**Race**,** n (%)**						
White	8,496 (80%)	2,605 (86%)	1,379 (80%)	3,277 (78%)	1,060 (77%)	175 (79%)
Arab or Middle East	19 (0.2%)	10 (0.3%)	1 (< 0.1%)	6 (0.1%)	2 (0.1%)	0 (0%)
Asian	1,641 (15%)	300 (9.8%)	277 (16%)	787 (19%)	248 (18%)	29 (13%)
Black or Afro-Caribbean	180 (1.7%)	77 (2.5%)	31 (1.8%)	42 (1.0%)	20 (1.5%)	10 (4.5%)
Others	258 (2.4%)	54 (1.8%)	42 (2.4%)	112 (2.7%)	42 (3.1%)	8 (3.6%)
**BMI**,** kg/m**^**2**^						
Mean (SD)	28.92 (6.02)	33.12 (6.60)	29.18 (5.02)	27.02 (4.53)	25.58 (4.58)	26.01 (5.53)
Median (25%, 75%)	27.80 (24.80,32.00)	32.10 (28.20,37.10)	28.35 (25.73,31.90)	26.40 (24.00,29.40)	25.00 (22.50,28.10)	25.40 (22.30,28.30)
**Smoking status**,** n (%)**						
Never smoked	6,043 (57%)	1,476 (48%)	983 (57%)	2,554 (60%)	896 (65%)	134 (60%)
Ex-smoker	3,549 (34%)	1,129 (37%)	589 (34%)	1,347 (32%)	407 (30%)	77 (35%)
Current smoker	1,002 (9.5%)	441 (14%)	158 (9.1%)	323 (7.6%)	69 (5.0%)	11 (5.0%)
**Alcohol status**,** n (%)**						
No alcohol	4,525 (43%)	1,029 (34%)	672 (39%)	1,934 (46%)	766 (56%)	124 (56%)
< 1 drink/week	2,808 (27%)	870 (29%)	457 (26%)	1,080 (26%)	337 (25%)	64 (29%)
1–7 drinks/week	2,442 (23%)	820 (27%)	431 (25%)	941 (22%)	219 (16%)	31 (14%)
>=8 drinks/week	819 (7.7%)	327 (11%)	170 (9.8%)	269 (6.4%)	50 (3.6%)	3 (1.4%)
**Type of AF**,** n (%)**						
Paroxysmal AF	5,894 (56%)	1,660 (54%)	991 (57%)	2,349 (56%)	766 (56%)	128 (58%)
Persistent AF	3,712 (35%)	1,161 (38%)	586 (34%)	1,458 (35%)	440 (32%)	67 (30%)
Permanent AF	988 (9.3%)	225 (7.4%)	153 (8.8%)	417 (9.9%)	166 (12%)	27 (12%)
**CHA**_**2**_ **DS**_**2**_ **-VASc score**						
Mean (SD)	3.20 (1.48)	2.47 (1.25)	2.90 (1.36)	3.44 (1.39)	4.31 (1.35)	4.33 (1.46)
Medians (25%, 75%)	3.00 (2.00, 4.00)	2.00 (1.00, 3.00)	3.00 (2.00, 4.00)	3.00 (2.00, 4.00)	4.00 (3.00, 5.00)	4.00 (3.00, 5.00)
**Comorbidities**,** n (%)**						
Hypertension	8,083 (76%)	2,395 (79%)	1,293 (75%)	3,111 (74%)	1,093 (80%)	191 (86%)
Diabetes	2,542 (24%)	915 (30%)	364 (21%)	833 (20%)	358 (26%)	72 (32%)
Hyperlipidemia	4,519 (43%)	1,301 (43%)	734 (42%)	1,754 (42%)	609 (44%)	121 (55%)
Chronic artery disease	1,887 (18%)	458 (15%)	293 (17%)	771 (18%)	299 (22%)	66 (30%)
Congestive heart failure	2,337 (22%)	646 (21%)	342 (20%)	861 (20%)	409 (30%)	79 (36%)
Thromboembolism	1,579 (15%)	325 (11%)	225 (13%)	715 (17%)	272 (20%)	42 (19%)
Stroke	1,125 (11%)	236 (7.7%)	157 (9.1%)	498 (12%)	199 (15%)	35 (16%)
TIA	505 (4.8%)	109 (3.6%)	77 (4.5%)	223 (5.3%)	89 (6.5%)	7 (3.2%)
Previous bleeding	574 (5.4%)	121 (4.0%)	82 (4.7%)	256 (6.1%)	95 (6.9%)	20 (9.0%)
Peripheral artery disease	305 (2.9%)	67 (2.2%)	35 (2.0%)	126 (3.0%)	61 (4.4%)	16 (7.2%)
COPD	696 (6.6%)	181 (5.9%)	114 (6.6%)	253 (6.0%)	124 (9.0%)	24 (11%)
**Antiplatelet drug use**,** n (%)**	1,940 (18%)	561 (18%)	296 (17%)	758 (18%)	271 (20%)	54 (24%)
**Chronic dialysis**,** n (%)**	27 (0.3%)	0 (0%)	0 (0%)	0 (0%)	2 (0.1%)	25 (11%)
**Renal transplantation**,** n (%)**	15 (0.1%)	1 (< 0.1%)	1 (< 0.1%)	3 (< 0.1%)	7 (0.5%)	3 (1.4%)

Continuous variables were presented by Mean (SD) and Median (IQR). Catalogue variables were presented by frequency and percentage(n%)

CrCl, creatinine clearance (mL/min); BMI: body mass index, OAC: oral anticoagulation, VKA: Vitamin K antagonists, SD: standard deviation, IQR: interquartile range, ACE-I: angiotensin-converting enzyme inhibitors, ARB: angiotensin II receptor blockers, TIA: Transient ischemic attack, COPD: chronic obstructive pulmonary disease

Patients with CrCl 30 to < 50 mL/min were the oldest (79.22 ± 6.21 years) while patients with CrCl > 95 mL/min had the highest mean BMI (33.12 ± 6.60 kg/m^2^). For patients with CrCl < 30 mL/min, 51% received VKA and 28% received apixaban, with the latter having the highest CHA_2_DS_2_-VASc score (4.33 ± 1.46).

### 3-year cumulative incidence of clinical adverse results

After three years of follow-up, NOAC users had lower cumulative incidence rates of all-cause death (6.48% vs.10.35%, *P* < 0.01), composite outcomes (11.33% vs. 15.36%, *P* < 0.001), CV death (2.82% vs. 4.55%, *P* < 0.001), and major bleeding (3.70% vs. 5.07%, *P* = 0.001) compared with VKA therapy (Table 2).

**Table 2:** Three-year cumulative incidence rate of different clinical adverse events in two different OACs cohorts (VKA or NOAC therapy) and 5 levels of renal function

Outcomes	Population divided by CrCl (mL/min)	VKA, *n* (3-year cumulative incidence rate%)	NOAC, *n* (3-year cumulative incidence rate%)	*P* value
All cause death	Overall	296 (10.35%)	501 (6.48%)	**< 0.001**
	> 95	42 (5.79%)	79 (3.40%)	**0.004**
	80 to < 95	31 (6.86%)	63 (4.93%)	0.12
	50 to < 80	97 (8.46%)	197 (6.40%)	**0.020**
	30 to < 50	81 (19.24%)	140 (14.72%)	**0.036**
	< 30	45 (39.47%)	22 (20.37%)	**0.002**
Composite outcomes	Overall	439 (15.36%)	876 (11.33%)	**< 0.001**
	> 95	72 (9.93%)	165 (7.11%)	**0.013**
	80 to < 95	48 (10.62%)	115 (9.00%)	0.3
	50 to < 80	160 (13.95%)	369 (11.99%)	0.087
	30 to < 50	108 (25.65%)	197 (20.72%)	**0.042**
	< 30	51 (44.74%)	30 (27.78%)	**0.009**
Cardiovascular death	Overall	130 (4.55%)	218 (2.82%)	**< 0.001**
	> 95	12 (1.66%)	33 (1.42%)	0.6
	80 to < 95	17 (3.76%)	27 (2.11%)	0.056
	50 to < 80	37 (3.23%)	80 (2.60%)	0.3
	30 to < 50	38 (9.03%)	67 (7.05%)	0.2
	< 30	26 (22.81%)	11 (10.19%)	**0.012**
Major bleeding	Overall	145 (5.07%)	286 (3.70%)	**0.001**
	> 95	31 (4.28%)	59 (2.54%)	**0.016**
	80 to < 95	14 (3.10%)	44 (3.44%)	0.7
	50 to < 80	55 (4.80%)	127 (4.13%)	0.3
	30 to < 50	33 (7.84%)	45 (4.73%)	**0.022**
	< 30	12 (10.53%)	11 (10.19%)	> 0.9
Myocardial infarction	Overall	53 (1.85%)	145 (1.87%)	> 0.9
	> 95	10 (1.38%)	28 (1.21%)	0.7
	80 to < 95	5 (1.11%)	19 (1.49%)	0.6
	50 to < 80	19 (1.66%)	63 (2.05%)	0.4
	30 to < 50	15 (3.56%)	30 (3.15%)	0.7
	< 30	4 (3.51%)	5 (4.63%)	0.7
Stroke	Overall	78 (2.73%)	190 (2.46%)	0.4
	> 95	15 (2.07%)	41 (1.77%)	0.6
	80 to < 95	10 (2.21%)	21 (1.64%)	0.4
	50 to < 80	32 (2.79%)	84 (2.73%)	> 0.9
	30 to < 50	15 (3.56%)	39 (4.10%)	0.6
	< 30	6 (5.26%)	5 (4.63%)	0.8
TE	Overall	81 (2.83%)	216 (2.79%)	> 0.9
	> 95	15 (2.07%)	44 (1.90%)	0.8
	80 to < 95	11 (2.43%)	28 (2.19%)	0.8
	50 to < 80	31 (2.70%)	94 (3.05%)	0.5
	30 to < 50	16 (3.80%)	44 (4.63%)	0.5
	< 30	8 (7.02%)	6 (5.56%)	0.7

CrCl, creatinine clearance; TE, thromboembolism

For patients with CrCl > 95 mL/min, participants receiving NOAC had a lower cumulative incidence of all-cause death (3.40% vs. 5.79%, *P* = 0.004), composite outcomes (7.11% vs. 9.93%, *P* = 0.013) and major bleeding (2.54% vs. 4.28%, *P* = 0.016). For patients with CrCl < 30 mL/min, patients prescribed NOAC had a lower cumulative incidence rate of all-cause death (20.37% vs. 39.47%, *P* = 0.002), composite outcomes (27.78% vs. 44.74%, *P* = 0.009) and CV death (10.19% vs. 22.81%, *P* = 0.012).

### Multivariable nonlinear analysis and RCS curves for renal function

**Fig. 1:**
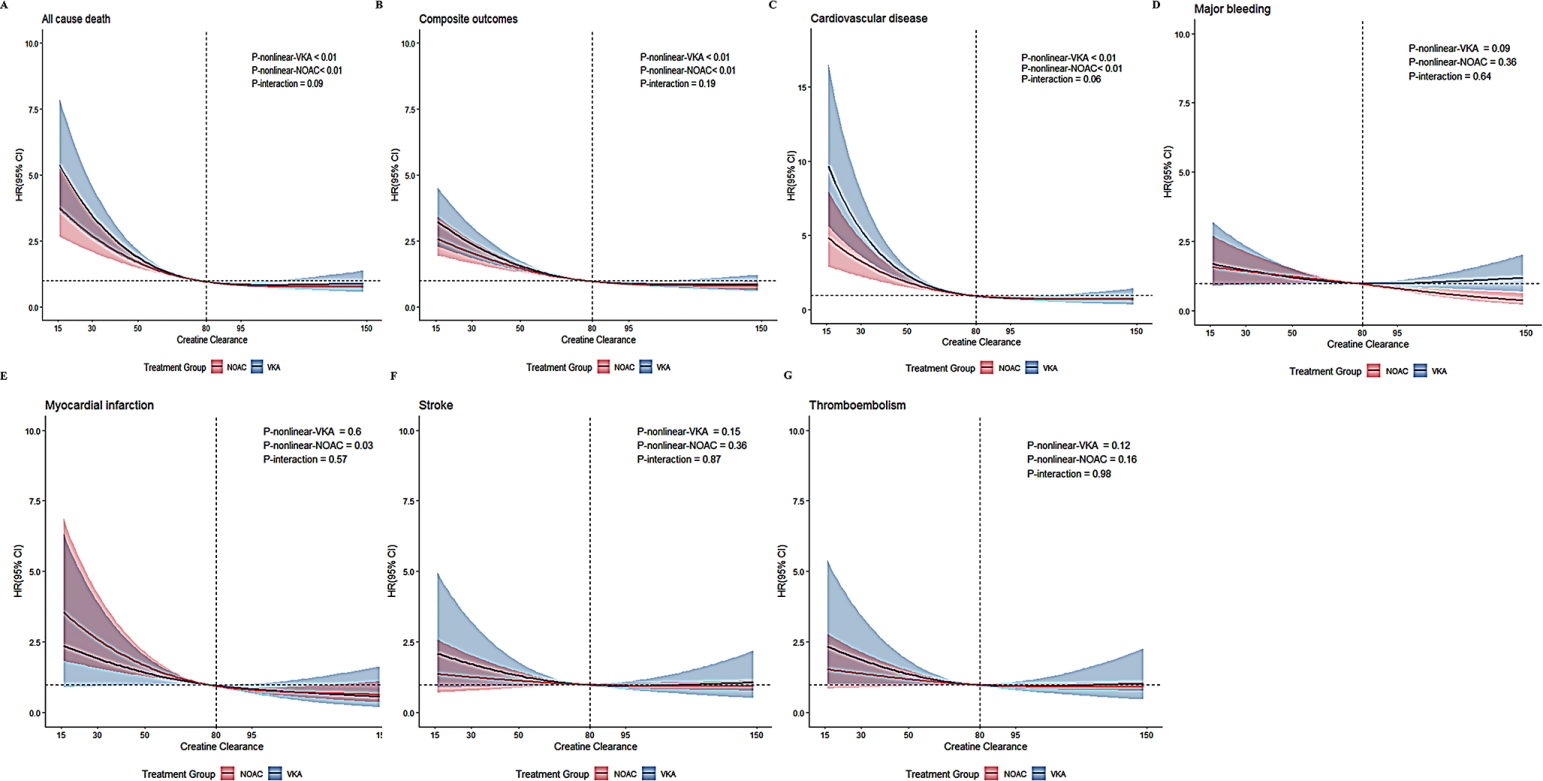
Restrictive cubic spline curve. VKA, vitamin K anticoagulant; Ref., reference; NOACs, Non-vitamin K antagonist oral anticoagulants

After adjustment, the estimated RCS curve suggested nonlinear associations in outcomes of all-cause death, composite outcomes, and CV death in VKA and NOAC groups with an ‘L-shaped’ curve. For patients with CrCl < 80 mL/min, the risk of all-cause death, composite outcomes and CV death declined with increasing CrCl in patients with VKA/NOAC therapy. For patients with CrCl > 80 mL/min, those prescribed NOAC had lower risk of major bleeding compared to those prescribed a VKA (Fig. 1).

**Fig. 2:**
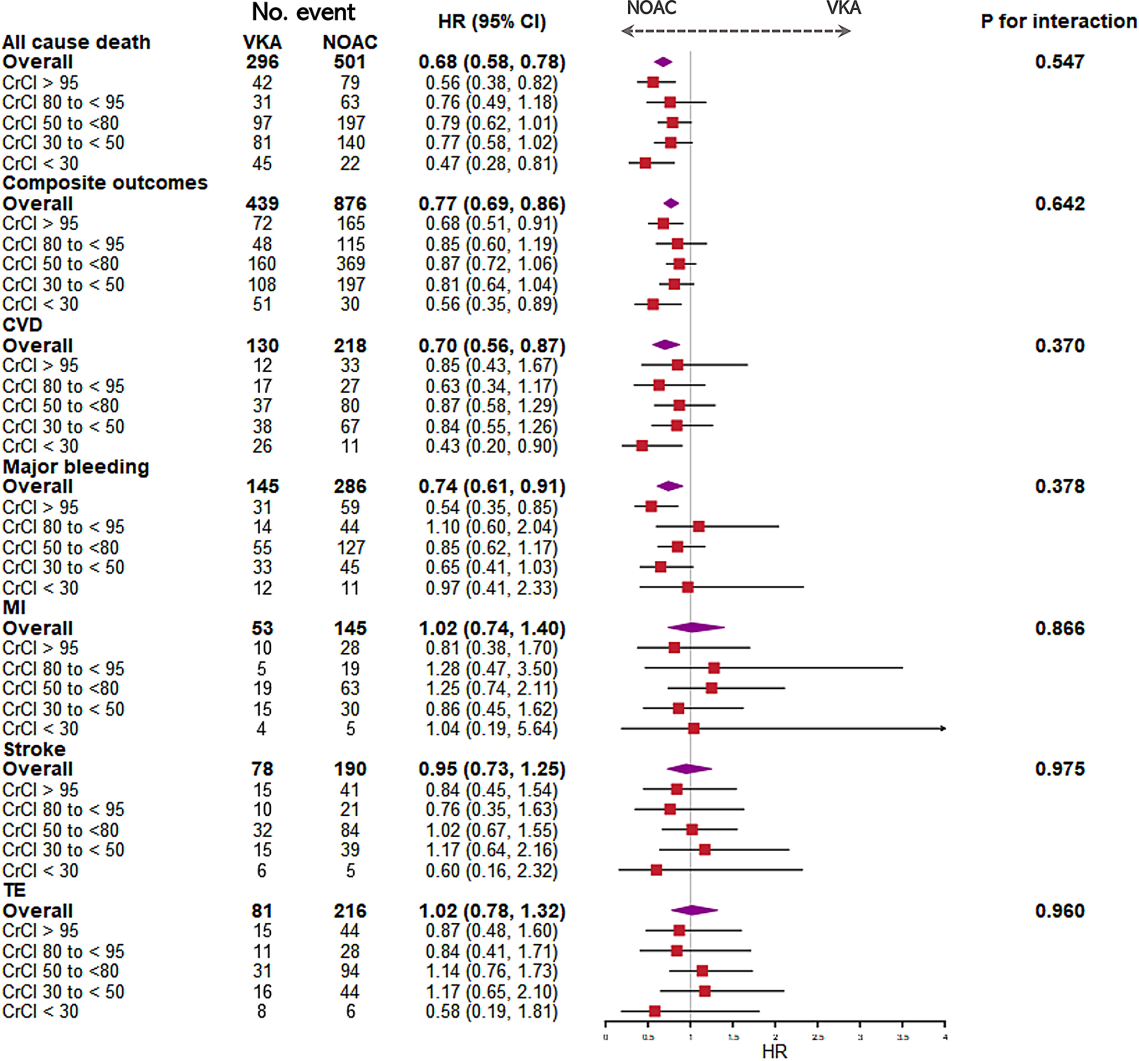
Forest plot of analysis of risk of clinical end events in AF patients of different creatinine clearance by cox regression comparing NOAC vs. VKA (reference) treatment. Models were adjusted by age, sex, race (Asian /not Asian), BMI, smoking and drinking status, history of hypertension, cardiovascular disease, congestive heart failure, history of thromboembolism, chronic obstructive pulmonary disease, peripheral artery disease, diabetes, previous bleeding, and any antiplatelet drug use. CrCl (mL/min): creatinine clearance; No., number of events; HR: hazard ratio; CI, confidence intervals; VKA, vitamin K anticoagulant; Ref., reference; NOACs, Non-vitamin K antagonist oral anticoagulants; CVD: cardiovascular death; MI: myocardial infarction; TE, thromboembolism events

### Univariable and multivariable analysis for OAC type

Figure 2 shows the forest plot of the Cox regression analysis. Considering all AF patients, those prescribed NOAC had lower risk of all-cause death (HR 0.62, 95% CI 0.54–0.71), composite outcomes (HR 0.72, 95% CI 0.65–0.81), CV death (HR 0.62, 95% CI 0.50–0.76) and major bleeding (HR 0.72, 95% CI 0.59–0.88) compared to those prescribed VKA.

Patients prescribed with NOAC consistently had lower risk of all-cause death at every CrCl groups. For those with CrCl < 30 mL/min, NOAC prescription was associated with lower risk of all-cause death (HR: 0.48, 95% CI: 0.29–0.80), composite outcomes (HR 0.58, 95% CI 0.37–0.90), CV death (HR: 0.42, 95% CI: 0.21–0.85). After adjustment for age, sex, BMI, smoking/drinking status, comorbidities and pharmacotherapies, similar results were found in patients with CrCl > 95 mL/min and CrCl < 30 mL/min.

**Table 3:** Analysis of risk of clinical end events in AF patients of different creatinine clearance by cox regression comparing individual NOACs vs. VKA (reference) treatment

Outcomes	No.	HR (95% CI)	*P*	No.	HR (95% CI)	*P*	No.	HR (95% CI)	*P*	No.	HR (95% CI)	*P*	NO.	HR (95% CI)	*P*	No.	HR (95% CI)	*P*	*P* for interaction
	All			CrCl > 95 mL/min			CrCl 80 to < 95 mL/min			CrCl 50 to < 80 mL/min			CrCl 30 to < 50 mL/min			CrCl < 30 mL/min			
**All cause death**																			
VKA	296	Ref.		42	Ref.		31	Ref.		97	Ref.		81	Ref.		45	Ref.		0.429
Apixaban	206	**0.69** **(0.57**,** 0.82)**	**< 0.001**	34	0.70 (0.44, 1.11)	0.129	22	0.67 (0.38, 1.16)	0.150	76	0.80 (0.59, 1.08)	0.150	62	**0.70** **(0.50**,** 0.99)**	**0.041**	**12**	**0.44** **(0.23**,** 0.85)**	**0.015**	
Dabigatran	123	**0.63** **(0.51**,** 0.78)**	**< 0.001**	16	**0.46** **(0.26**,** 0.82)**	**0.009**	23	1.00 (0.58, 1.74)	> 0.9	57	0.80 (0.58, 1.12)	0.200	25	**0.63** **(0.40**,** 1.00)**	**0.050**	**2**	**-**		
Rivaroxaban	168	**0.73** **(0.60**,** 0.88)**	**0.001**	28	**0.50** **(0.31**,** 0.82)**	**0.006**	18	0.71 (0.39, 1.29)	0.300	62	0.81 (0.59, 1.12)	0.200	52	1.00 (0.70, 1.43)	> 0.9	8	0.86 (0.38, 1.93)	0.700	
**Composite outcomes**																			
VKA	439	Ref.		72	Ref.		48	Ref.		160	Ref.		108	Ref.		51	Ref.		0.387
Apixaban	354	**0.79** **(0.68**,** 0.91)**	**< 0.001**	63	0.73 (0.51, 1.03)	0.073	42	0.82 (0.54, 1.25)	0.400	143	0.90 (0.71, 1.13)	0.400	87	0.75 (0.56, 1.00)	0.054	19	0.63 (0.36, 1.10)	0.100	
Dabigatran	215	**0.70** **(0.60**,** 0.83)**	**< 0.001**	42	0.7 (0.47, 1.02)	0.065	33	0.82 (0.53, 1.29)	0.400	100	0.81 (0.63, 1.05)	0.110	38	0.72 (0.49, 1.06)	0.095	2	-	**-**	
Rivaroxaban	293	**0.82** **(0.71**,** 0.95)**	**0.010**	56	**0.61** **(0.43**,** 0.87)**	**0.006**	40	0.97 (0.63, 1.48)	0.900	118	0.91 (0.71, 1.16)	0.400	70	1.00 (0.74, 1.36)	> 0.9	9	0.78 (0.36, 1.69)	0.500	
**Cardiovascular death**																			
VKA	130	Ref.		12	Ref.		17	Ref.		37	Ref.		38	Ref.		26	Ref.		0.411
Apixaban	84	**0.67** **(0.51**,** 0.89)**	**0.006**	15	1.08 (0.49, 2.37)	0.846	10	0.59 (0.27, 1.32)	0.200	27	0.74 (0.44, 1.23)	0.200	28	0.73 (0.44, 1.20)	0.200	4	**0.31** **(0.10**,** 0.91)**	**0.034**	
Dabigatran	54	**0.66** **(0.48**,** 0.91)**	**0.012**	6	0.66 (0.25, 1.79)	0.418	8	0.63 (0.27, 1.50)	0.300	24	0.98 (0.58, 1.66)	> 0.9	15	0.91 (0.49, 1.69)	0.800	1	-	-	
Rivaroxaban	78	0.79 (0.60, 1.06)	0.112	12	0.74 (0.33, 1.69)	0.480	9	0.74 (0.32, 1.70)	0.500	27	0.91 (0.55, 1.51)	0.700	24	1.01 (0.60, 1.70)	> 0.9	6	0.91 (0.33, 2.46)	0.800	
**Major bleeding**																			
VKA	145	Ref.		31	Ref.		14	Ref.		55	Ref.		33	Ref.		12	Ref.		0.196
Apixaban	107	**0.71** **(0.55**,** 0.91)**	**0.008**	19	**0.48** **(0.27**,** 0.86)**	**0.014**	15	1.05 (0.50, 2.20)	0.900	50	0.87 (0.59, 1.29)	0.500	16	**0.48** **(0.26**,** 0.90)**	**0.021**	7	1.20 (0.45, 3.20)	0.700	
Dabigatran	58	**0.57** **(0.42**,** 0.77)**	**< 0.001**	15	0.55 (0.30, 1.03)	0.060	9	0.71 (0.30, 1.67)	0.400	27	0.65 (0.41, 1.03)	0.065	7	0.48 (0.21, 1.09)	0.080	0	-	-	
Rivaroxaban	113	0.91 (0.71, 1.17)	0.475	23	**0.57** **(0.33**,** 0.99)**	**0.047**	20	1.70 (0.84, 3.43)	0.140	44	0.94 (0.63, 1.40)	0.800	22	1.07 (0.61, 1.85)	0.800	4	1.21 (0.33, 4.46)	0.800	
**MI**																			
VKA	53	Ref.		10	Ref.		5	Ref.		19	Ref.		15	Ref.		4	Ref.		0.757
Apixaban	66	1.16 (0.80, 1.67)	0.434	12	1.04 (0.44, 2.47)	0.929	7	1.2 (0.37, 3.90)	0.800	27	1.33 (0.73, 2.42)	0.400	16	0.94 (0.45, 1.95)	0.900	4	1.45 (0.22, 9.41)	0.700	
Dabigatran	29	0.81 (0.51, 1.27)	0.358	6	0.75 (0.27, 2.11)	0.590	6	1.4 (0.42, 4.71)	0.600	12	0.90 (0.43, 1.86)	0.800	4	0.58 (0.19, 1.79)	0.300	1	-	-	
Rivaroxaban	47	1.01 (0.68, 1.50)	0.957	8	0.55 (0.21, 1.44)	0.226	6	1.33 (0.39, 4.50)	0.600	23	1.49 (0.80, 2.76)	0.200	10	0.94 (0.41, 2.15)	0.900	0	-	-	
**Stroke**																			
VKA	78	Ref.		15	Ref.		10	Ref.		32	Ref.		15	Ref.		6	Ref.		0.546
Apixaban	76	1.00 (0.72, 1.38)	0.997	15	0.85 (0.40, 1.80)	0.677	8	0.82 (0.31, 2.12)	0.700	37	1.24 (0.76, 2.02)	0.400	14	0.92 (0.44, 1.93)	0.800	2	0.45 (0.08, 2.70)	0.400	
Dabigatran	56	0.98 (0.69, 1.38)	0.888	13	0.99 (0.47, 2.11)	0.986	8	0.88 (0.34, 2.26)	0.800	23	0.90 (0.52, 1.54)	0.700	12	1.62 (0.75, 3.53)	0.200	0	-	-	
Rivaroxaban	54	0.89 (0.63, 1.26)	0.517	11	0.62 (0.28, 1.38)	0.242	5	0.62 (0.21, 1.85)	0.400	23	0.96 (0.56, 1.65)	0.900	12	1.22 (0.57, 2.63)	0.600	3	2.12 (0.38, 11.9)	0.400	
**TE**																			
VKA	81	Ref.		15	Ref.		11	Ref.		31	Ref.		16	Ref.		8	Ref.		0.550
Apixaban	90	1.07 (0.79, 1.46)	0.643	17	0.91 (0.44, 1.88)	0.81	13	1.08 (0.48, 2.47)	0.85	39	1.26 (0.78, 2.05)	0.35	17	0.95 (0.48, 1.92)	0.90	4	0.83 (0.22, 3.10)	0.786	
Dabigatran	60	1.00 (0.72, 1.41)	0.983	14	1.05 (0.50, 2.20)	0.90	7	0.62 (0.24, 1.63)	0.34	25	1.00 (0.59, 1.71)	0.99	14	1.64 (0.78, 3.44)	0.19	0	-	0.999	
Rivaroxaban	62	0.96 (0.69, 1.35)	0.831	11	0.62 (0.28, 1.36)	0.23	8	0.88 (0.35, 2.23)	0.79	29	1.22 (0.73, 2.04)	0.45	12	1.14 (0.53, 2.43)	0.74	2	0.94 (0.15, 5.92)	0.948	

Models were adjusted by age, sex, race (Asian /not Asian), BMI, smoking and drinking status, history of hypertension, cardiovascular disease, congestive heart failure, history of thromboembolism, chronic obstructive pulmonary disease, peripheral artery disease, diabetes, previous bleeding, and any antiplatelet drug use

HR, hazard ratio; CI, confidence intervals; No., number of events; CrCl, creatinine clearance; MI, myocardial infarction; TE, thromboembolism; BMI, body mass index; VKA, vitamin K anticoagulant; Ref., reference;

### Individual NOACs versus VKA

Comparisons of individual OAC drugs are shown in Table 3. Given the small number of patients prescribed edoxaban (CrCl at all levels) and dabigatran (CrCl < 30 mL/min), these data were not analysed. Compared to a VKA, apixaban and dabigatran were associated with a decreased risk of all-cause death (HR: 0.69, 95% CI: 0.57–0.82) (HR: 0.63, 95% CI: 0.51–0.78) and composite outcomes (HR: 0.79, 95% CI: 0.68–0.91) (HR: 0.70, 95%CI: 0.60–0.83).

Compared to a VKA, apixaban was consistently associated with a decreased risk of all-cause death in patients with CrCl = 30–50 and < 30 mL/min. Dabigatran was associated with a lower risk of all cause death in patients with CrCl > 95 and 30–50 mL/min. Rivaroxaban was associated with decreased risk of all-cause death, composite outcomes, and major bleeding in patients with CrCl > 95mL/min.

### Age subgroups

The baseline table suggested that patients with lower renal function were older, and were more likely to receive VKA. Therefore, we explored the impact of age on outcomes with NOAC versus VKA. Supplementary Fig. 1 shows the age subgroup of association between outcomes and NOAC versus VKA. Patients receiving NOAC were associated with a lower risk of major bleeding in patients with age ≥ 75 (HR: 0.60, 95% CI: 0.46–0.80, P_interaction_ = 0.042). No significant interaction was noted between age groups and OAC use in other outcomes (All P_interaction_ > 0.05).

### Association between Asian/Non-Asian individuals and clinical events

Previous studies have shown that Asian patients are prescribed NOAC less frequently [15] and have a higher risk of bleeding [16]. In our baseline, we observed significant heterogeneity in renal function among Asian patients. Therefore, we performed the analysis to assess the outcome risks in Asian patients with varying renal functions (Supplementary Fig. 2). Non-Asian individuals were associated with higher risk of all-cause death (HR: 1.51, 95% CI: 1.17–1.93), composite outcomes (HR: 1.38, 95% CI: 1.15–1.66), and major bleeding (HR: 1.46, 95% CI: 1.05–2.03). No significant interaction was noted between ethnic groups and renal function (All P_interaction_ >0.05).

## Discussion

We analysed the impact of renal function, measured by CrCl, on major clinical events in patients prescribed VKAs and NOACs. Our finding indicated that increasing CrCl was associated with decreased risk of all-cause death, composite outcomes, and CV death among patients with CrCl < 80mL/min. Furthermore, NOAC use was independently correlated with a lower risk of all-cause death, composite outcomes, CV death, and major bleeding compared to VKA. Additionally, NOACs were consistently associated with decreased risk of all-cause death and composite outcomes across different levels of renal function (with a more pronounced effect in patients with CrCl > 95 and < 30 ml/min). No significant interaction was observed between OAC therapy and renal function on all-cause death. These results reinforce the importance of evaluating renal function when prescribing anticoagulants and highlight the benefits of NOAC in patients with diverse renal profiles.

Worsening CrCl has been identified as an independent predictor of ischemic stroke, systemic embolism, and bleeding in patients with AF [17]. In the ROCKET AF subgroup analysis, CrCl was analyzed as a continuous variable, revealing that the HR of all stroke and systemic embolism risk increased by 12% for every 10 mL/min decline in renal function (HR, 1.12; 95% CI, 1.07–1.16), irrespective of OAC use [18]. In contrast, our study found no significant association between decreasing CrCl and the risk of stroke, likely because our cohort included only patients receiving OACs (with 70% oarticipants receiving NOACs).

For patients with AF and stage 3 CKD who are at elevated risk of stroke, warfarin, direct thrombin or factor Xa inhibitors are the recommended treatment options. For those with stage 4 CKD, treatment with warfarin or labelled doses of NOAC is also considered reasonable (class 2a recommendation) to reduce the risk of stroke [3]. When prescribing OACs for CKD patients, it is essential to carefully balance the risk of thromboembolism against the risk of bleeding [19], particularly in the Asian population who may have an evaluated bleeding risks [16]. Indeed, CKD patients with AF often present with clinical complexities, including a high prevalence of frailty, multimorbidity and polypharmacy, which underscores the need for a more holistic and individualized treatment approach [20, 21].

Consistent with our study, the ARISTOTLE trial demonstrated that apixaban significantly reduced the risk of major bleeding compared to VKA, but with a decreasing trend among patients with a CrCl of 25–50 ml/min [22]. In contrast, the ORBIT AF study showed no interaction between OAC therapy and CKD concerning the risk of all-cause mortality and CV death after adjusting for covariates [23].

For patients with severe renal dysfunction, the present study demonstrated NOAC prescription was associated with reduced risks of all-cause death, and CV death, but not with the risk of major bleeding. Dabigatran significantly reduced the risk of CV death and major bleeding, and the same reductions were observed for apixaban, but not for rivaroxaban in patients with CrCl 30 to < 50 mL/min.

Previous studies demonstrated similar findings. Coccheri et al. found that dabigatran was better than VKA for avoiding major bleeding; the number needed to treat (NNT) was lower in patients treated with dabigatran than with VKA. Data concerning apixaban, and rivaroxaban showed higher NNT [24]. Dabigatran is generally not recommended for use in patients with CrCl < 30 mL/min, although our study showed a small number of AF patients with CrCl < 30 mL/min used dabigatran. In patients with severe CKD, our data indicated that apixaban prescription was associated with better safety versus VKA, but other NOACs were not associated with better safety. Apixaban is less dependent on renal excretion compared with other NOACs, which could explain its superior safety benefit [25]. A meta-analysis indicated that compared to dabigatran, apixaban was associated with less major bleeding in patients with moderate renal impairment (CrCl 25–49 ml/min) [26] which is consistent with our result in patients with moderate renal impairment (CrCl 30 to < 50 ml/min). Also, previous studies demonstrated a slower decline in renal function in patients taking NOAC compared with warfarin [27].

Patients with high CrCl (> 95mL/min) were more likely to benefit from NOACs compared to VKAs, particularly regarding the risks of all-cause death, major bleeding, and the composite outcome. Dabigatran, apixaban and rivaroxaban demonstrated similar risks of clinical outcomes. Similarly, Korean nationwide data showed that dabigatran, apixaban, and rivaroxaban were safer than warfarin in AF patients with CrCl > 95 mL/min [28]. Korean cohort data also showed that edoxaban was associated with a decreased risk of major bleeding and mortality during a median follow-up period of 5 months [29]. A US *post-hoc* analysis showed that among patients with CrCl > 80mL/min, the risks of stroke and bleeding rates were similar among warfarin users and dabigatran users, but the risk of first ischemic stroke was lower in dabigatran users (HR 0.84), and the risk was higher in rivaroxaban user and apixaban user compared to warfarin users (HRs 1.07 and 1.35, respectively) [30]. On the other hand, in patients with CrCl > 80 mL/min from the ROCKET-AF trial, the risk of a composite of stroke and systemic embolism was not statistically significant between warfarin and rivaroxaban users, aligning with our study findings [31]. Similarly, in the ENGAGE AF-TIMI 48 study, high-dose edoxaban users did not exhibit a significantly higher risk of stroke or systemic embolism compared to warfarin users (HR: 1.36, 95%CI: 0.88–2.10) in patients with CrCl > 95 mL/min [32].

Dabigatran, apixaban, and rivaroxaban demonstrated similar risks for clinical outcomes. Consistent with these findings, Korean nationwide data indicated that dabigatran, apixaban, and rivaroxaban were safer than warfarin in AF patients with CrCl > 95 mL/min [28]. Additionally, Korean cohort data revealed that edoxaban was associated with a reduced risk of major bleeding and mortality during a median follow-up period of 5 months [29].

In a US post-hoc analysis, among patients with CrCl > 80 mL/min, stroke and bleeding risks were similar between warfarin and dabigatran users; however, the risk of first ischemic stroke was lower in dabigatran users (HR 0.84) and higher in rivaroxaban and apixaban users compared to warfarin users (HRs 1.07 and 1.35, respectively) [30]. Conversely, data from the ROCKET-AF trial showed no statistically significant difference in the risk of stroke or systemic embolism between VKA and NOAC users in patients with CrCl > 80 mL/min, aligning with our study findings. Similarly, the ENGAGE AF-TIMI 48 study found that high-dose edoxaban users did not have a significantly higher risk of stroke or systemic embolism compared to warfarin users (HR: 1.36, 95% CI: 0.88–2.10) in patients with CrCl > 95 mL/min.

## Strengths and limitations

Our study has several strengths. First, using the global GLORIA-AF registry, we included a large cohort of “real life” AF patients with detailed 3-years of follow-up, and with over 70% NOAC prescription rate. Also, the registry includes patients with impaired renal function (CrCl < 30 mL/min) and with normal renal function (CrCl > 95mL/min).

However, there are limitations. First, this study is a *post-hoc* retrospective analysis with limited data to characterize the renal function before the onset of atrial fibrillation, which may have affected the risk of the observed outcomes. Second, although we extensively adjusted our results with multivariable Cox regression analyses, we did not include some important dependent factors, such as the CKD treatment, the anticoagulation regimes, and other co-medications which may lead to increased risk of major bleeding [33]. Third, the number of patients prescribed edoxaban was limited, as well as those with a CrCl < 30 mL/min for each individual NOAC, particular reducing the statistical power for some of our observations and comparisons. Additionally, OAC use was influenced by physician choice, patient adherence, and treatment management strategies. Therefore, our result should be interpreted with caution.

## Conclusion

In this large prospective global registry, NOAC prescription was associated with better outcomes than VKA, regardless of renal function. These findings highlight the importance of considering renal function when choosing OAC and demonstrate the advantages of NOACs across different renal profiles. It may be advisable for clinicians to prioritize NOACs over VKAs, particularly in patients with impaired renal function or CrCl outside the 30–95 mL/min range, where the benefits were most significant.

## Electronic supplementary material

Below is the link to the electronic supplementary material.

Supplementary Material 1
