# Supplementary material for: Renal function and adverse clinical events in anticoagulated patients with atrial fibrillation: insights from the GLORIA-AF Registry Phase III
Source: J Thromb Thrombolysis. 2025 Feb 9;58(2):165–77. doi: 10.1007/s11239-025-03067-5 (PMC11885355; doi:10.1007/s11239-025-03067-5)
Supplement: Supplementary file 1 — Supplementary Material 1 [file 11239_2025_3067_MOESM1_ESM.docx]

**Renal Function and Adverse Clinical Events in Anticoagulated Patients with Atrial Fibrillation: Insights from the GLORIA-AF Registry Phase III**

Yang Liu^1,2^, Steven Ho Man Lam^1*^, Giulio Francesco Romiti^1,4^, Bi Huang^1,3^, Yang Chen^1^, Tze Fan Chao^5,6^, Brian Olshansky^7^, Kui Hong^2,8,9^, Menno V. Huisman ^10†^, Gregory Y. H. Lip^1,11 †^, *on behalf of the GLORIA-AF Investigators*

**Supplementary Materials**

**[Appendix – List of GLORIA-AF Investigators](#_Toc180684102)** [2](#_Toc180684102)

**[Supplementary figure 1: Subgroup analysis of association between Asian/Non-Asian and outcomes in different level of CrCl](#_Toc180684103)** [8](#_Toc180684103)

**[Supplementary figure 2: Age subgroup analysis of association between outcomes and NOAC versus VKA](#_Toc180684104)** [9](#_Toc180684104)

# Appendix – List of GLORIA-AF Investigators

| Dzifa Wosornu Abban | Bouziane Benhalima | Jei Keon Chae |
| --- | --- | --- |
| Nasser Abdul | Jutta Bergler-Klein | Kathrine Chalamidas |
| Atilio Marcelo Abud | Jean-Baptiste Berneau | Krishnan Challappa |
| Fran Adams | Richard A. Bernstein | Sunil Prakash Chand |
| Srinivas Addala | Percy Berrospi | Harinath Chandrashekar |
| Pedro Adragão | Sergio Berti | Ludovic Chartier |
| Walter Ageno | Andrea Berz | Kausik Chatterjee |
| Rajesh Aggarwal | Elizabeth Best | Carlos Antero Chavez Ayala |
| Sergio Agosti | Paulo Bettencourt | Aamir Cheema |
| Piergiuseppe Agostoni | Robert Betzu | Amjad Cheema |
| Francisco Aguilar | Ravi Bhagwat | Lin Chen |
| Julio Aguilar Linares | Luna Bhatta | Shih-Ann Chen |
| Luis Aguinaga | Francesco Biscione | Jyh Hong Chen |
| Jameel Ahmed | Giovanni Bisignani | Fu-Tien Chiang |
| Allessandro Aiello | Toby Black | Francesco Chiarella |
| Paul Ainsworth | Michael J. Bloch | Lin Chih-Chan |
| Jorge Roberto Aiub | Stephen Bloom | Yong Keun Cho |
| Raed Al-Dallow | Edwin Blumberg | Jong-Il Choi |
| Lisa Alderson | Mario Bo | Dong Ju Choi |
| Jorge Antonio Aldrete Velasco | Ellen Bøhmer | Guy Chouinard |
| Dimitrios Alexopoulos | Andreas Bollmann | Danny Hoi-Fan Chow |
| Fernando Alfonso Manterola | Maria Grazia Bongiorni | Dimitrios Chrysos |
| Pareed Aliyar | Giuseppe Boriani | Galina Chumakova |
| David Alonso | D.J. Boswijk | Eduardo Julián José Roberto Chuquiure Valenzuela |
| Fernando Augusto Alves da Costa | Jochen Bott | Nicoleta Cindea Nica |
| José Amado | Edo Bottacchi | David J. Cislowski |
| Walid Amara | Marica Bracic Kalan | Anthony Clay |
| Mathieu Amelot | Drew Bradman | Piers Clifford |
| Nima Amjadi | Donald Brautigam | Andrew Cohen |
| Fabrizio Ammirati | Nicolas Breton | Michael Cohen |
| Marianna Andrade | P.J.A.M. Brouwers | Serge Cohen |
| Nabil Andrawis | Kevin Browne | Furio Colivicchi |
| Giorgio Annoni | Jordi Bruguera Cortada | Ronan Collins |
| Gerardo Ansalone | A. Bruni | Paolo Colonna |
| M.Kevin Ariani | Claude Brunschwig | Steve Compton |
| Juan Carlos Arias | Hervé Buathier | Derek Connolly |
| Sébastien Armero | Aurélie Buhl | Alberto Conti |
| Chander Arora | John Bullinga | Gabriel Contreras Buenostro |
| Muhammad Shakil Aslam | Jose Walter Cabrera | Gregg Coodley |
| M. Asselman | Alberto Caccavo | Martin Cooper |
| Philippe Audouin | Shanglang Cai | Julian Coronel |
| Charles Augenbraun | Sarah Caine | Giovanni Corso |
| S. Aydin | Leonardo Calò | Juan Cosín Sales |
| Ivaneta Ayryanova | Valeria Calvi | Yves Cottin |
| Emad Aziz | Mauricio Camarillo Sánchez | John Covalesky |
| Luciano Marcelo Backes | Rui Candeias | Aurel Cracan |
| E. Badings | Vincenzo Capuano | Filippo Crea |
| Ermentina Bagni | Alessandro Capucci | Peter Crean |
| Seth H. Baker | Ronald Caputo | James Crenshaw |
| Richard Bala | Tatiana Cárdenas Rizo | Tina Cullen |
| Antonio Baldi | Francisco Cardona | Harald Darius |
| Shigenobu Bando | Francisco Carlos da Costa Darrieux | Patrick Dary |
| Subhash Banerjee | Yan Carlos Duarte Vera | Olivier Dascotte |
| Alan Bank | Antonio Carolei | Ira Dauber |
| Gonzalo Barón Esquivias | Susana Carreño | Vicente Davalos |
| Craig Barr | Paula Carvalho | Ruth Davies |
| Maria Bartlett | Susanna Cary | Gershan Davis |
| Vanja Basic Kes | Gavino Casu | Jean-Marc Davy |
| Giovanni Baula | Claudio Cavallini | Mark Dayer |
| Steffen Behrens | Guillaume Cayla | Marzia De Biasio |
| Alan Bell | Aldo Celentano | Silvana De Bonis |
| Raffaella Benedetti | Tae-Joon Cha | Raffaele De Caterina |
| Juan Benezet Mazuecos | Kwang Soo Cha | Teresiano De Franceschi |
| J.R. de Groot | William French | Christian Hall |
| José De Horta | Keith Friedman | Bing Han |
| Axel De La Briolle | Athena Friese | Seongwook Han |
| Gilberto de la Pena Topete | Ana Gabriela Fruntelata | Joe Hargrove |
| Angelo Amato Vicenzo de Paola | Shigeru Fujii | David Hargroves |
| Weimar de Souza | Stefano Fumagalli | Kenneth B. Harris |
| A. de Veer | Marta Fundamenski | Tetsuya Haruna |
| Luc De Wolf | Yutaka Furukawa | Emil Hayek |
| Eric Decoulx | Matthias Gabelmann | Jeff Healey |
| Sasalu Deepak | Nashwa Gabra | Steven Hearne |
| Pascal Defaye | Niels Gadsbøll | Michael Heffernan |
| Freddy Del-Carpio Munoz | Michel Galinier | Geir Heggelund |
| Diana Delic Brkljacic | Anders Gammelgaard | J.A. Heijmeriks |
| N. Joseph Deumite | Priya Ganeshkumar | Maarten Hemels |
| Silvia Di Legge | Christopher Gans | I. Hendriks |
| Igor Diemberger | Antonio Garcia Quintana | Sam Henein |
| Denise Dietz | Olivier Gartenlaub | Sung-Ho Her |
| Pedro Dionísio | Achille Gaspardone | Paul Hermany |
| Qiang Dong | Conrad Genz | Jorge Eduardo Hernández Del Río |
| Fabio Rossi dos Santos | Frédéric Georger | Yorihiko Higashino |
| Elena Dotcheva | Jean-Louis Georges | Michael Hill |
| Rami Doukky | Steven Georgeson | Tetsuo Hisadome |
| Anthony D'Souza | Evaldas Giedrimas | Eiji Hishida |
| Simon Dubrey | Mariusz Gierba | Etienne Hoffer |
| Xavier Ducrocq | Ignacio Gil Ortega | Matthew Hoghton |
| Dmitry Dupljakov | Eve Gillespie | Kui Hong |
| Mauricio Duque | Alberto Giniger | Suk keun Hong |
| Dipankar Dutta | Michael C. Giudici | Stevie Horbach |
| Nathalie Duvilla | Alexandros Gkotsis | Masataka Horiuchi |
| A. Duygun | Taya V. Glotzer | Yinglong Hou |
| Rainer Dziewas | Joachim Gmehling | Jeff Hsing |
| Charles B. Eaton | Jacek Gniot | Chi-Hung Huang |
| William Eaves | Peter Goethals | David Huckins |
| L.A Ebels-Tuinbeek | Seth Goldbarg | kathy Hughes |
| Clifford Ehrlich | Ronald Goldberg | A. Huizinga |
| Sabine Eichinger-Hasenauer | Britta Goldmann | E.L. Hulsman |
| Steven J. Eisenberg | Sergey Golitsyn | Kuo-Chun Hung |
| Adnan El Jabali | Silvia Gómez | Gyo-Seung Hwang |
| Mahfouz El Shahawy | Juan Gomez Mesa | Margaret Ikpoh |
| Mauro Esteves Hernandes | Vicente Bertomeu Gonzalez | Davide Imberti |
| Ana Etxeberria Izal | Jesus Antonio Gonzalez Hermosillo | Hüseyin Ince |
| Rudolph Evonich III | Víctor Manuel González López | Ciro Indolfi |
| Oksana Evseeva | Hervé Gorka | Shujiro Inoue |
| Andrey Ezhov | Charles Gornick | Didier Irles |
| Raed Fahmy | Diana Gorog | Harukazu Iseki |
| Quan Fang | Venkat Gottipaty | C. Noah Israel |
| Ramin Farsad | Pascal Goube | Bruce Iteld |
| Laurent Fauchier | Ioannis Goudevenos | Venkat Iyer |
| Stefano Favale | Brett Graham | Ewart Jackson-Voyzey |
| Maxime Fayard | G. Stephen Greer | Naseem Jaffrani |
| Jose Luis Fedele | Uwe Gremmler | Frank Jäger |
| Francesco Fedele | Paul G. Grena | Martin James |
| Olga Fedorishina | Martin Grond | Sung-Won Jang |
| Steven R. Fera | Edoardo Gronda | Nicolas Jaramillo |
| Luis Gustavo Gomes Ferreira | Gerian Grönefeld | Nabil Jarmukli |
| Jorge Ferreira | Xiang Gu | Robert J. Jeanfreau |
| Claudio Ferri | Ivett Guadalupe Torres Torres | Ronald D. Jenkins |
| Anna Ferrier | Gabriele Guardigli | Carlos Jerjes Sánchez |
| Hugo Ferro | Carolina Guevara | Javier Jimenez |
| Alexandra Finsen | Alexandre Guignier | Robert Jobe |
| Brian First | Michele Gulizia | Tomas Joen-Jakobsen |
| Stuart Fischer | Michael Gumbley | Nicholas Jones |
| Catarina Fonseca | Albrecht Günther | Jose Carlos Moura Jorge |
| Luísa Fonseca Almeida | Andrew Ha | Bernard Jouve |
| Steven Forman | Georgios Hahalis | Byung Chun Jung |
| Brad Frandsen | Joseph Hakas | Kyung Tae Jung |
| Werner Jung | Torben Larsen | Nolan Mayer |
| Mikhail Kachkovskiy | Karine Lavandier | John McClure |
| Krystallenia Kafkala | Jessica LeBlanc | Terry McCormack |
| Larisa Kalinina | Moon Hyoung Lee | William McGarity |
| Bernd Kallmünzer | Chang-Hoon Lee | Hugh McIntyre |
| Farzan Kamali | John Lehman | Brent McLaurin |
| Takehiro Kamo | Ana Leitão | Feliz Alvaro Medina Palomino |
| Priit Kampus | Nicolas Lellouche | Francesco Melandri |
| Hisham Kashou | Malgorzata Lelonek | Hiroshi Meno |
| Andreas Kastrup | Radoslaw Lenarczyk | Dhananjai Menzies |
| Apostolos Katsivas | T. Lenderink | Marco Mercader |
| Elizabeth Kaufman | Salvador León González | Christian Meyer |
| Kazuya Kawai | Peter Leong-Sit | Beat j. Meyer |
| Kenji Kawajiri | Matthias Leschke | Jacek Miarka |
| John F. Kazmierski | Nicolas Ley | Frank Mibach |
| P Keeling | Zhanquan Li | Dominik Michalski |
| José Francisco Kerr Saraiva | Xiaodong Li | Patrik Michel |
| Galina Ketova | Weihua Li | Rami Mihail Chreih |
| AJIT Singh Khaira | Xiaoming Li | Ghiath Mikdadi |
| Aleksey Khripun | Christhoh Lichy | Milan Mikus |
| Doo-Il Kim | Ira Lieber | Davor Milicic |
| Young Hoon Kim | Ramon Horacio Limon Rodriguez | Constantin Militaru |
| Nam Ho Kim | Hailong Lin | Sedi Minaie |
| Dae Kyeong Kim | Gregory Y. H. Lip | Bogdan Minescu |
| Jeong Su Kim | Feng Liu | Iveta Mintale |
| June Soo Kim | Hengliang Liu | Tristan Mirault |
| Ki Seok Kim | Guillermo Llamas Esperon | Michael J. Mirro |
| Jin bae Kim | Nassip Llerena Navarro | Dinesh Mistry |
| Elena Kinova | Eric Lo | Nicoleta Violeta Miu |
| Alexander Klein | Sergiy Lokshyn | Naomasa Miyamoto |
| James J. Kmetzo | Amador López | Tiziano Moccetti |
| G. Larsen Kneller | José Luís López-Sendón | Akber Mohammed |
| Aleksandar Knezevic | Adalberto Menezes Lorga Filho | Azlisham Mohd Nor |
| Su Mei Angela Koh | Richard S. Lorraine | Michael Mollerus |
| Shunichi Koide | Carlos Alberto Luengas | Giulio Molon |
| Anastasios Kollias | Robert Luke | Sergio Mondillo |
| J.A. Kooistra | Ming Luo | Patrícia Moniz |
| Jay Koons | Steven Lupovitch | Lluis Mont |
| Martin Koschutnik | Philippe Lyrer | Vicente Montagud |
| William J. Kostis | Changsheng Ma | Oscar Montaña |
| Dragan Kovacic | Genshan Ma | Cristina Monti |
| Jacek Kowalczyk | Irene Madariaga | Luciano Moretti |
| Natalya Koziolova | Koji Maeno | Kiyoo Mori |
| Peter Kraft | Dominique Magnin | Andrew Moriarty |
| Johannes A. Kragten | Gustavo Maid | Jacek Morka |
| Mori Krantz | Sumeet K. Mainigi | Luigi Moschini |
| Lars Krause | Konstantinos Makaritsis | Nikitas Moschos |
| B.J. Krenning | Rohit Malhotra | Andreas Mügge |
| F. Krikke | Rickey Manning | Thomas J. Mulhearn |
| Z. Kromhout | Athanasios Manolis | Carmen Muresan |
| Waldemar Krysiak | Helard Andres Manrique Hurtado | Michela Muriago |
| Priya Kumar | Ioannis Mantas | Wlodzimierz Musial |
| Thomas Kümler | Fernando Manzur Jattin | Carl W. Musser |
| Malte Kuniss | Vicky Maqueda | Francesco Musumeci |
| Jen-Yuan Kuo | Niccolo Marchionni | Thuraia Nageh |
| Achim Küppers | Francisco Marin Ortuno | Hidemitsu Nakagawa |
| Karla Kurrelmeyer | Antonio Martín Santana | Yuichiro Nakamura |
| Choong Hwan Kwak | Jorge Martinez | Toru Nakayama |
| Bénédicte Laboulle | Petra Maskova | Gi-Byoung Nam |
| Arthur Labovitz | Norberto Matadamas Hernandez | Michele Nanna |
| Wen Ter Lai | Katsuhiro Matsuda | Indira Natarajan |
| Andy Lam | Tillmann Maurer | Hemal M. Nayak |
| Yat Yin Lam | Ciro Mauro | Stefan Naydenov |
| Fernando Lanas Zanetti | Erik May | Jurica Nazlić |
| Charles Landau | Torben Larsen | Alexandru Cristian Nechita |
| Giancarlo Landini | Karine Lavandier | Libor Nechvatal |
| Estêvão Lanna Figueiredo | Jessica LeBlanc | Sandra Adela Negron |
| James Neiman | Arnold Pinter | Hamdi Sati |
| Fernando Carvalho Neuenschwander | Fausto Pinto | Irina Savelieva |
| David Neves | R. Pisters | Pierre-Jean Scala |
| Anna Neykova | Nediljko Pivac | Peter Schellinger |
| Ricardo Nicolás Miguel | Darko Pocanic | Carlos Scherr |
| George Nijmeh | Cristian Podoleanu | Lisa Schmitz |
| Alexey Nizov | Alessandro Politano | Karl-Heinz Schmitz |
| Rodrigo Noronha Campos | Zdravka Poljakovic | Bettina Schmitz |
| Janko Nossan | Stewart Pollock | Teresa Schnabel |
| Tatiana Novikova | Jose Polo Garcéa | Steffen Schnupp |
| Ewa Nowalany-Kozielska | Holger Poppert | Peter Schoeniger |
| Emmanuel Nsah | Maurizio Porcu | Norbert Schön |
| Juan Carlos Nunez Fragoso | Antonio Pose Reino | Peter Schwimmbeck |
| Svetlana Nurgalieva | Neeraj Prasad | Clare Seamark |
| Dieter Nuyens | Dalton Bertolim Précoma | Greg Searles |
| Ole Nyvad | Alessandro Prelle | Karl-Heinz Seidl |
| Manuel Odin de Los Rios Ibarra | John Prodafikas | Barry Seidman |
| Philip O'Donnell | Konstantin Protasov | Jaroslaw Sek |
| Martin O'Donnell | Maurice Pye | Lakshmanan Sekaran |
| Seil Oh | Zhaohui Qiu | Carlo Serrati |
| Yong Seog Oh | Jean-Michel Quedillac | Neerav Shah |
| Dongjin Oh | Dimitar Raev | Vinay Shah |
| Gilles O'Hara | Carlos Antonio Raffo Grado | Anil Shah |
| Kostas Oikonomou | Sidiqullah Rahimi | Shujahat Shah |
| Claudia Olivares | Arturo Raisaro | Vijay Kumar Sharma |
| Richard Oliver | Bhola Rama | Louise Shaw |
| Rafael Olvera Ruiz | Ricardo Ramos | Khalid H. Sheikh |
| Christoforos Olympios | Maria Ranieri | Naruhito Shimizu |
| Anna omaszuk-Kazberuk | Nuno Raposo | Hideki Shimomura |
| Joaquín Osca Asensi | Eric Rashba | Dong-Gu Shin |
| eena Padayattil jose | Ursula Rauch-Kroehnert | Eun-Seok Shin |
| Francisco Gerardo Padilla Padilla | Ramakota Reddy | Junya Shite |
| Victoria Padilla Rios | Giulia Renda | Gerolamo Sibilio |
| Giuseppe Pajes | Shabbir Reza | Frank Silver |
| A. Shekhar Pandey | Luigi Ria | Iveta Sime |
| Gaetano Paparella | Dimitrios Richter | Tim A. Simmers |
| F Paris | Hans Rickli | Narendra Singh |
| Hyung Wook Park | Werner Rieker | Peter Siostrzonek |
| Jong Sung Park | Tomas Ripolil Vera | Didier Smadja |
| Fragkiskos Parthenakis | Luiz Eduardo Ritt | David W. Smith |
| Enrico Passamonti | Douglas Roberts | Marcelo Snitman |
| Rajesh J. Patel | Ignacio Rodriguez Briones | Dario Sobral Filho |
| Jaydutt Patel | Aldo Edwin Rodriguez Escudero | Hassan Soda |
| Mehool Patel | Carlos Rodríguez Pascual | Carl Sofley |
| Janice Patrick | Mark Roman | Adam Sokal |
| Ricardo Pavón Jimenez | Francesco Romeo | Yannie Soo Oi Yan |
| Analía Paz | E. Ronner | Rodolfo Sotolongo |
| Vittorio Pengo | Jean-Francois Roux | Olga Ferreira de Souza |
| William Pentz | Nadezda Rozkova | Jon Arne Sparby |
| Beatriz Pérez | Miroslav Rubacek | Jindrich Spinar |
| Alma Minerva Pérez Ríos | Frank Rubalcava | David Sprigings |
| Alejandro Pérez-Cabezas | Andrea M. Russo | Alex C. Spyropoulos |
| Richard Perlman | Matthieu Pierre Rutgers | Dimitrios Stakos |
| Viktor Persic | Karin Rybak | Clemens Steinwender |
| Francesco Perticone | Samir Said | Georgios Stergiou |
| Terri K. Peters | Tamotsu Sakamoto | Ian Stiell |
| Sanjiv Petkar | Abraham Salacata | Marcus Stoddard |
| Luis Felipe Pezo | Adrien Salem | Anastas Stoikov |
| Christian Pflücke | Rafael Salguero Bodes | Witold Streb |
| David N. Pham | Marco A. Saltzman | Ioannis Styliadis |
| Roland T. Phillips | Alessandro Salvioni | Guohai Su |
| Stephen Phlaum | Gregorio Sanchez Vallejo | Xi Su |
| Denis Pieters | Marcelo Sanmartín Fernández | Wanda Sudnik |
| Kai Sukles | Alberta L. Warner | Tiziana Tassinari |
| Julien Pineau | Wladmir Faustino Saporito | Ashis Tayal |
| Jens Taggeselle | Takeshi Yamashita |  |
| Yuichiro Takagi | Ping Yen Bryan Yan |  |
| Amrit Pal Singh Takhar | Tianlun Yang |  |
| Angelika Tamm | Yoto Yotov |  |
| Katsumi Tanaka | Ralf Zahn |  |
| Tanyanan Tanawuttiwat | Stuart Zarich |  |
| Sherman Tang | Sergei Zenin |  |
| Aylmer Tang | Elisabeth Louise Zeuthen |  |
| Giovanni Tarsi | Huanyi Zhang |  |
| Emmanuel Touze | Donghui Zhang |  |
| Elina Trendafilova | Xingwei Zhang |  |
| W. Kevin Tsai | Kouki Watanabe |  |
| Hung Fat Tse | Jeanne Wei |  |
| Hiroshi Tsutsui | Christian Weimar |  |
| Tian Ming Tu | Stanislav Weiner |  |
| Ype Tuininga | Renate Weinrich |  |
| Minang Turakhia | Ming-Shien Wen |  |
| Samir Turk | Marcus Wiemer |  |
| Wayne Turner | Preben Wiggers |  |
| Arnljot Tveit | Andreas Wilke |  |
| Richard Tytus | Ping Zhang |  |
| C Valadão | Jun Zhang |  |
| P.F.M.M. van Bergen | Shui Ping Zhao |  |
| Philippe van de Borne | Yujie Zhao |  |
| B.J. van den Berg | Zhichen Zhao |  |
| C van der Zwaan | Yang Zheng |  |
| M. Van Eck | Jing Zhou |  |
| Peter Vanacker | Sergio Zimmermann |  |
| Dimo Vasilev | Andrea Zini |  |
| Vasileios Vasilikos | Steven Zizzo |  |
| Maxim Vasilyev | Wenxia Zong |  |
| Srikar Veerareddy | L Steven Zukerman |  |
| Mario Vega Miño | Muzahir Tayebjee |  |
| Asok Venkataraman | J.M. ten Berg |  |
| Paolo Verdecchia | Dan Tesloianu |  |
| Francesco Versaci | Salem H.K. The |  |
| Ernst Günter Vester | Dierk Thomas |  |
| Hubert Vial | Serge Timsit |  |
| Jason Victory | Tetsuya Tobaru |  |
| Alejandro Villamil | Andrzej R. Tomasik. |  |
| Marc Vincent | Mikhail Torosoff |  |
| Anthony Vlastaris |  |  |
| Jürgen vom Dahl |  |  |
| Kishor Vora |  |  |
| Robert B. Vranian |  |  |
| Paul Wakefield |  |  |
| Ningfu Wang |  |  |
| Mingsheng Wang |  |  |
| Xinhua Wang |  |  |
| Feng Wang |  |  |
| Tian Wang |  |  |
| David Williams |  |  |
| Marcus L. Williams |  |  |
| Bernhard Witzenbichler |  |  |
| Brian Wong |  |  |
| Ka Sing Lawrence Wong |  |  |
| Beata Wozakowska-Kaplon |  |  |
| Shulin Wu |  |  |
| Richard C. Wu |  |  |
| Silke Wunderlich |  |  |
| Nell Wyatt |  |  |
| John (Jack) Wylie |  |  |
| Yong Xu |  |  |
| Xiangdong Xu |  |  |
| Hiroki Yamanoue |  |  |

**
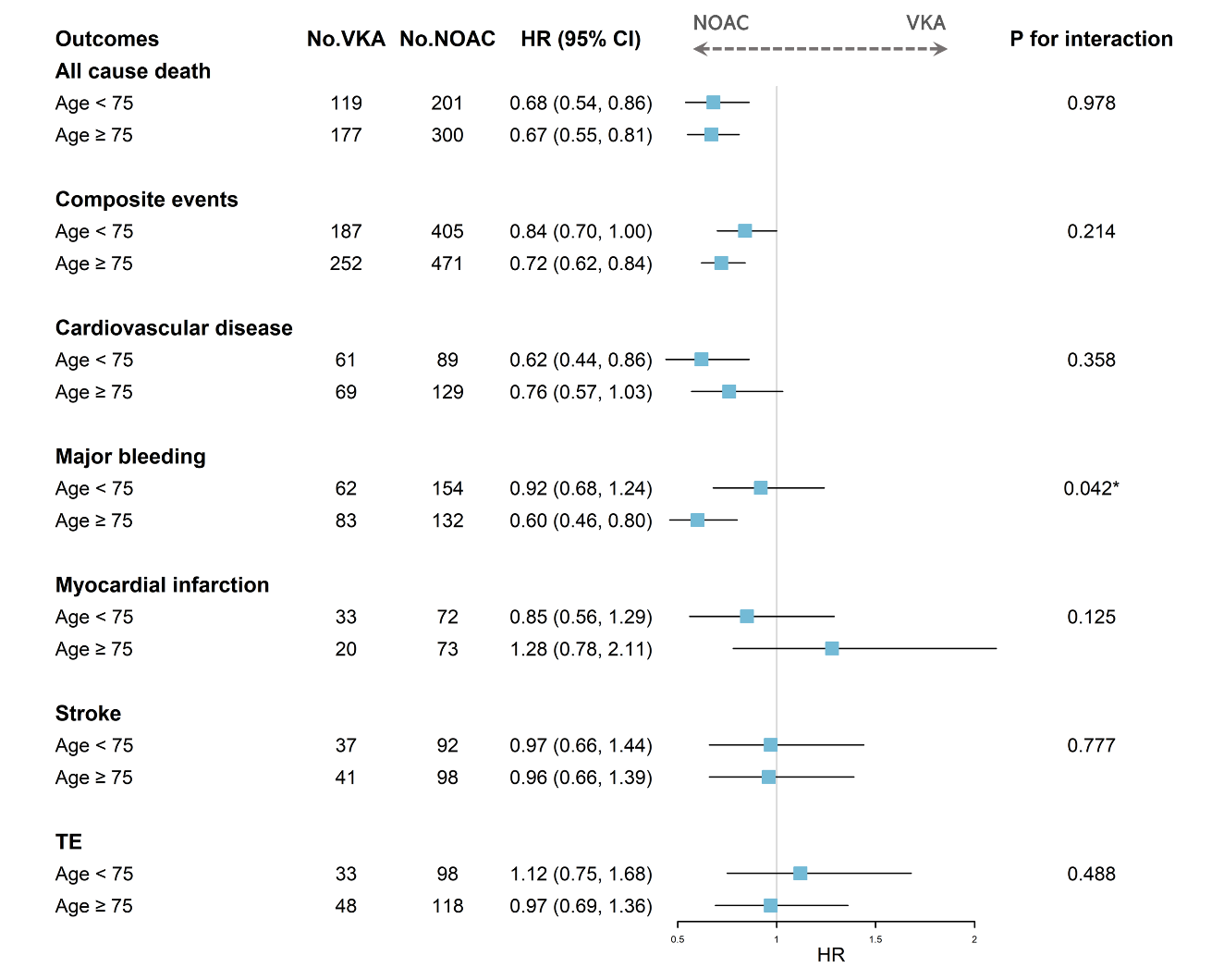
**

# Supplementary figure 1: Age subgroup analysis of association between outcomes and NOAC versus VKA

* indicates Pinteraction < 0.05

Models were adjusted by age, sex, race (Asian /not Asian), BMI, smoking and drinking status, history of hypertension, cardiovascular disease, congestive heart failure, history of TE, chronic obstructive pulmonary disease, peripheral artery disease, diabetes, previous bleeding, and any antiplatelet drug use.

Composite outcomes included all-cause death, major bleeding and TE.

No., number of events; TE, thromboembolism events; BMI, body mass index; VKA, vitamin K anticoagulant; NOACs, Non-vitamin K antagonist oral anticoagulants;


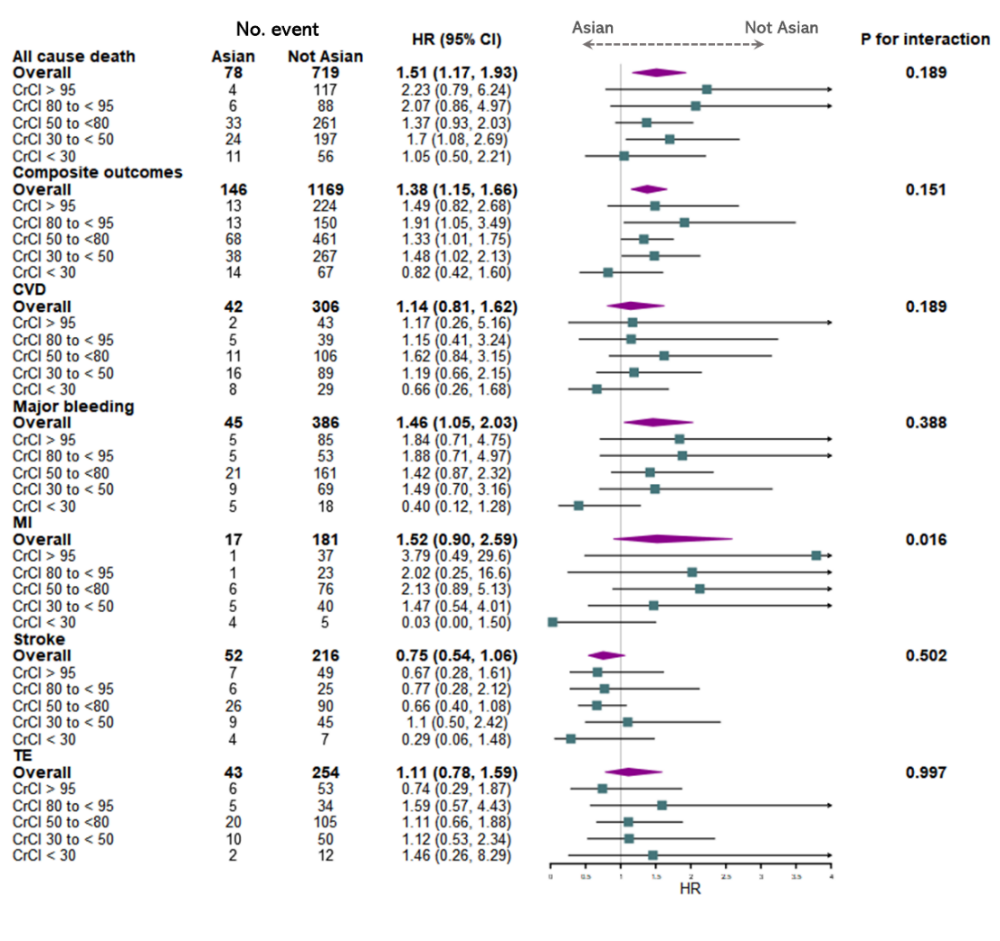


# Supplementary figure 2: Subgroup analysis of association between Asian/Non-Asian and outcomes in different level of CrCl

Models were adjusted by age, sex, race (Asian /not Asian), BMI, smoking and drinking status, history of hypertension, cardiovascular disease, congestive heart failure, history of thromboembolism, chronic obstructive pulmonary disease, peripheral artery disease, diabetes, previous bleeding, and any antiplatelet drug use.

CrCl (mL/min): creatinine clearance; No., number of events; HR: hazard ratio; CI, confidence intervals; CVD: cardiovascular death; MI: myocardial infarction; TE, thromboembolism events;
